# Working Memory and Language Contribution to Verbal Learning and Memory in Drug-Resistant Unilateral Focal Temporal Lobe Epilepsy

**DOI:** 10.3389/fneur.2021.780086

**Published:** 2021-12-08

**Authors:** Monica Bolocan, Claudia I. Iacob, Eugen Avram

**Affiliations:** Laboratory of Health Psychology and Clinical Neuropsychology, Department of Applied Psychology and Psychotherapy, Faculty of Psychology and Educational Sciences, University of Bucharest, Bucharest, Romania

**Keywords:** drug-resistant temporal lobe epilepsy, working memory, verbal memory and learning, language, picture naming

## Abstract

We aimed to investigate the working memory (WM) and language separate contributions to verbal learning and memory in patients with unilateral drug-resistant temporal lobe epilepsy (drTLE); additionally, we explored the mediating role of WM on the relationship between the number of antiepileptic drugs (AEDs) and short-term verbal memory. We retrospectively enrolled 70 patients with left (LTLE; *n* = 44) and right (RTLE; *n* = 26) drTLE. About 40 similar (age and education) healthy controls were used to determine impairments of groups at WM, language (naming and verbal fluency), and verbal learning and memory (five trials list-learning, story memory—immediate recall). To disentangle the effect of learning from the short-term memory, we separately analyzed performances at the first trial, last trial, and delayed-recall list-learning measures, in addition to the total learning capacity (the sum of the five trials). Correlation and regression analyses were used to assess the contribution of potential predictors while controlling for main clinical and demographic variables, and ascertain the mediating role of WM. All patients were impaired at WM and story memory, whereas only LTLE showed language and verbal learning deficits. In RTLE, language was the unique predictor for the most verbal learning performances, whereas WM predicted the results at story memory. In LTLE, WM was the sole predictor for short-term verbal learning (list-learning capacity; trial 1) and mediated the interaction between AED number and the performance at these measures, whereas language predicted the delayed-recall. Finally, WM confounded the performance at short-term memory in both groups, although at different measures. WM is impaired in drTLE and contributes to verbal memory and learning deficits in addition to language, mediating the relationship between AED number and short-term verbal memory in LTLE. Clinicians should consider this overlap when interpreting poor performance at verbal learning and memory in drTLE.

## Introduction

Patients with temporal lobe epilepsy show heterogeneous cognitive phenotypes, including various deficits not only in verbal memory and language (1, 2) but also in working memory (WM) (3). WM, a dedicated system for short-term active manipulation of the information, serves as a foundation for various cognitive processes, including verbal learning, language comprehension, and production (4, 5).

Although WM and the short-term verbal memory span concepts significantly overlap (6), they represent distinct entities (7), functionally correlated within a multi-store memory model (8). Short-term memory span refers to the retention of a limited number of data (in the verbal domain, roughly the George Miller's “magical” number 7 ± 2 items) for a relatively short time (usually up to 30 s) (9). Instead, WM involves an active organization and manipulation of the stored information and relates to executive functioning (4).

Findings regarding the WM impairment in drug-resistant temporal lobe epilepsy (drTLE) are discrepant (10–13), most probably due to the different complexity of the tasks used to measure WM. The WM capacity is commonly measured with complex supra-span tasks involving both storage and processing components (14), and it highly depends on the item semantic grouping and complexity (15). Thus, verbal memory tests used in the drTLE assessment, like list-learning and story-learning (16), are differently influenced by WM, having different demands on the semantic processing (17). Although the verbal memory and WM were found to have a high degree of overlap for both tasks (18), other findings suggest that an executive dysfunction impacts the list-learning task performance more than the story memory task (19). In particular, the unrelated list-learning task puts a heavy accent on the rote learning and is considered to be critically dependent on WM (20).

At the same time, language is a powerful predictor for verbal learning and memory (21, 22) and interferes with the verbal memory performance in language-dominant drTLE (23, 24). Therefore, language abilities must be considered when interpreting the verbal memory performance (17, 25), particularly in patients with word-finding difficulties (26).

The poor cognitive outcome is generally associated with an early onset and a long duration of the disease, and with poor seizure control (27, 28). In particular, seizure-related variables, like age at onset, disease duration, and seizure frequency, seem to be more critical than sociodemographic variables, like age and intelligence quotient (IQ), when interpreting results of neuropsychological tests (29). Nevertheless, polypharmacy induces attentional or arousal deficits (30), affecting executive functioning and broader neuropsychological functions (31–33), contributing to cumulating deficits in learning and memory. Executive function and memory showed higher negative correlations with the number of AEDs than with the defined daily dose; therefore, the AED number is a sensitive measure for side effects (34). Executive functions are particularly sensitive to drug load, whereas learning and memory are less vulnerable (35, 36); therefore, the relationship between polytherapy and verbal memory is most likely mediated by executive functions, including the WM capacity (33).

This study aims to understand the unique contributions of the verbal/auditory WM and language in various stages of the verbal learning and memory (i.e., short-term, delayed) of patients with left (LTLE) or right (RTLE) foci, beyond the influence of the primary demographic and disease characteristics. Additionally, we explored the mediating effect of WM on the relationship between the AED number and the short-term verbal memory. We expect the performance at the last list-learning trial and delayed-recall measures to be predicted mainly by language. An opposite pattern of predictors (WM as the main predictor) is expected for the first list-learning trial and story memory (immediate recall), both measures of the short-term verbal memory. We emphasize the importance of the integrated interpretation of the neuropsychological tests used in the pre-surgical assessment of patients with drTLE to unravel the reasons behind the poor verbal memory performance of a patient, thus improving the postoperative cognitive decline prediction and informing the rehabilitation programs.

## Materials and Methods

### Participants and Procedure

We retrospectively enrolled 70 consecutive patients with unilateral drTLE, further divided according to the seizure-onset lateralization into LTLE (*n* = 44) and RTLE (*n* = 26). Patients (right-handed adults, 55.71% male, age: M = 31.77, SD = 9.65 years; age at seizure onset: M = 20.66, SD = 11.69 years; disease duration: M = 10.80, SD = 9.43 years; IQ > 80) were candidates for epilepsy surgery and have been referred between 2019 and 2021 for neuropsychological evaluation by the epileptologists from the Epilepsy Monitoring Unit of the University Emergency Hospital Bucharest. The medical records of patients concluded the diagnosis of unilateral focal drTLE based on the following factors: case history, seizure semiology, interictal and/or ictal video-EEG, and brain imaging (1.5- or 3-T MRI, interictal [18F] fluorodeoxyglucose–PET). Exclusion criteria: moderate or severe mood disorder (e.g., anxiety, depression, and stress); bilateral TLE features; incomplete data. About 40 healthy right-handed individuals (50% female; age: M = 33.90, SD = 9.98) were recruited from the broader community to provide an age and education equivalent control sample to the patient sample. Exclusion criteria for the controls comprised any history of neurologic disease or major psychiatric illness, current psychoactive medication, and IQ < 80. Controls signed a consent form detailing the study procedure under the guidelines from the local ethical committee.

The sociodemographic, clinical, and neuropsychological characteristics of participants are summarized in Table 1. There are no significant differences between groups in sex, χ^2^(2) = 0.88, *p* = 0.645, ϕ = 0.09, age at assessment, χ^2^(2) = 3.09, *p* = 0.213, ϕ = 0.21, education years, χ^2^(2) = 1.63, *p* = 0.443, ϕ = 0.12, and IQ level, χ^2^(2) = 1.13, *p* = 0.568, ϕ = 0.10.

**Table 1:** Sociodemographic, clinical, and neuropsychological characteristics of participants.

	**LTLE**	**RTLE**	**Controls**	***p*-value**
	***n* = 44**	***n* = 26**	***n* = 40**	
Age				
y, mean ± SD	33.02 ± 9.92	29.65 ± 8.96	33.90 ± 9.98	0.209*
(range)	(18–57)	(18–49)	(18–56)	
Gender				
F, *n*, %	18 (41)	13 (50)	20 (50)	0.645*
Education				
y, mean ± SD	13.80 ± 2.29	13.65 ± 2.23	14.20 ± 2.28	0.546*
(range)	(11–19)	(11–18)	(11–19)	
IQ				
Inferior (80–89), *n*, %	10 (23)	7 (27)	10 (25)	0.568*
Average (90–110), *n*, %	21 (48)	15 (58)	19 (48)	
Superior (> 110), *n*, %	13 (30)	4 (15)	11 (28)	
Age at seizure onset			-	
y, mean ± SD	21.89 ± 11.39	17.35 ± 11.45		0.069*
(range)	(1–53)	(1–44)		
Duration of epilepsy			-	
y, mean ± SD	10.75 ± 8.70	11.54 ± 10.67		1.000*
(range)	(1–39)	(2–46)		
Seizure frequency / year			-	
mean ± SD	44.27 ± 99.29	133.73 ± 319.37		0.286*
(range)	(1–600)	(2–1500)		
HFC			-	
*n*, %	2 (5)	4 (15)		0.118*
History of TBI				
*n*, %	5 (11)	4 (15)	-	0.718*
History of FBTCS				
*n*, (%)	17 (39)	15 (58)	-	0.122*
No of AED				0.005*
mean ± SD	1.98 ± 0.73	2.54 ± 0.81	-	
(range)	(1–3)	(1–4)		
List-learning capacity				
mean ± SD	48.84 ± 8.96	50.62 ± 9.20	54.95 ± 5.86	0.001*
SE_M_	1.35	1.80	0.93	
List-learning trial 1				
mean ± SD	6.20 ± 1.75	6.62 ± 1.47	7.35 ± 1.03	0.004*
SE_M_	0.26	0.29	0.16	
List-learning trial 5				
mean ± SD	11.52 ± 2.44	12.42 ± 1.79	13.45 ± 0.93	<0.001*
SE_M_	0.37	0.35	0.15	
List-learning delayed-recall				
mean ± SD	9.20 ± 3.02	10.38 ± 3.03	11.88 ± 1.49	<0.001*
SE_M_	0.45	0.60	0.15	
Story memory				
mean ± SD	9.95 ± 4.05	11.31 ± 4.63	14.88 ± 2.74	<0.001*
SE_M_	0.61	0.91	0.43	
WM				
mean ± SD	9.48 ± 3.50	8.87 ± 3.46	12.07 ± 3.08	<0.001*
SE_M_	0.53	0.68	0.49	
Picture naming				
mean ± SD	28.91 ± 2.02	29.96 ± 1.46	30.57 ± 0.64	<0.001*
SE_M_	0.30	0.29	0.10	
Semantic fluency				
mean ± SD	19.43 ± 4.94	20.81 ± 5.80	23.52 ± 3.69	0.001**
SE_M_	0.74	1.14	0.58	
Phonological fluency				
mean ± SD	16.05 ± 4.00	16.27 ± 4.57	17.80 ± 3.46	0.106**
SE_M_	0.60	0.90	0.55	

IQ, intelligence quotient; LTLE, left drug-resistant temporal lobe epilepsy; RTLE, right drug-resistant temporal lobe epilepsy; HC, healthy controls; HFC, history of febrile convulsions; TBI, traumatic brain injury; FBTCS, focal to bilateral tonic-clonic seizures; AED, antiepileptic drugs; *nonparametric tests (Chi-square test, Kruskal–Wallis test, or Mann–Whitney U-test); **ANOVA with post-hoc Bonferroni corrections, alpha value of 0.05.

Patient groups show similar disease characteristics for epilepsy duration, *U* = 572, *z* = 0.00, *p* = 1.000, frequency of seizures per year, *U* = 484.5, *z* = −1.07, *p* = 0.286, history of febrile convulsions, χ^2^(1) = 2.45, *p* = 0.118, ϕ = 0.19, history of secondary generalized seizures, χ^2^(1) = 2.39, *p* = 0.122, ϕ = 0.18, history of encephalopathy, χ^2^(1) = 0.24, *p* = 0.627, ϕ = 0.06, and age at onset, *U* = 721.5, *z* = −1.82, *p* = 0.069, except higher number of AED in RTLE, *U* = 357.5, *z* = −2.78, *p* = 0.005.

### Measures

Verbal memory refers to storing verbally presented information, and tasks include learning word lists (i.e., list-learning) and short stories. The information can be recalled immediately after stimuli presentation or, usually, after 30 min (i.e., delayed recall) (37). Verbal memory was assessed with two tasks: five-trial unrelated list-learning and story memory (immediate recall). The unrelated list-learning test used in our study is an unpublished Romanian adaptation from Ray Auditory Verbal Learning test (38). The subject hears a list of 15 concrete, semantically unrelated words five times and, after each repetition, the subject is asked to immediately recall as many words as possible within 1 min, and freely recall the list after 30 min delay since the last repetition. To disentangle the effect of the short-term memory from learning, we analyzed the performance of patients in three stages of the list-learning test: trial 1—a measure of short-term verbal memory span; trial 5—a measure of learning ability; and delayed-recall, a measure of the retrieval capacity (39), and for the total number of correctly recalled items over the fifth trials (maximum 75), an indicator of the overall verbal learning capacity. The story memory test was an item of the Romanian Mini-Mental State Examination (MMSE)-−2 Expanded Version (40), where subjects must immediately repeat as accurately as possible a short story that is read to them (one trial). The number of correctly rendered keywords (maximum 25) is considered a measure of short-term semantic memory.

For the assessment of verbal/auditory WM, we selected an adapted version (41) of the Letter-Number Sequencing (LNS), a subtest of the fourth edition of the Wechsler Adult Intelligence Scale (42). Subjects listen to an auditory presentation of several series of mixed letters and numbers, increasing in length, and then reorder the numbers in ascending order and letters in alphabetical order, in increasing difficulty. LNS is a reliable complex span measure for WM used in both clinical and research settings (14, 43, 44).

Naming (or confrontation naming) is defined as the ability to label visually presented stimuli; the picture naming task requires the retrieval of the phonological and semantic information from the memory system (45). Naming abilities were assessed with the Neuropsychological Assessment Battery (NAB) Naming Test (46). The participants need to name the objects in a series of 31 colored pictures.

Verbal fluency is a cognitive function that facilitates information retrieval from memory, measured by asking subjects to retrieve specific information from a specific category or starting with a specific letter, usually within the 1-min time limit (47). Verbal fluency (phonemic and semantic) was assessed by asking participants to say as many words as possible within 1 min, starting with the letter “c,” respectively from the “animals” category. The letter “c” was chosen based on the analysis of the frequency of words in the Romanian dictionary as proven to reflect a high-frequency category of words.

Intelligence quotient was assessed with the RAVEN Progressive Matrices Standard test (48), a reliable measure of fluid intelligence. Raw scores were categorized as inferior (corresponding to an IQ range between 80 and 89), average (IQ range between 90 and 110), and superior (IQ > 110). The mood was assessed with Depression, Anxiety, Stress Scale (49), the 21 self-report items version, with the following cutoff scores: 13 for depression, 9 for anxiety, and 17 for stress.

### Data Analysis

Demographic characteristics were assessed using one-way univariate ANOVA with the groups (LTLE, RTLE, and controls) as between-subject factors. Patient groups were compared with *t*-tests or Mann–Whitney *U*-test on continuous clinical variables and chi-square on categorical variables.

We computed analyses in three steps. In the first step, we compared participant groups using ANOVA with *post-hoc* comparisons (Bonferroni corrections), based on an alpha of 0.05, supplemented with the Kruskal–Wallis test. The Spearman correlation analysis (alpha value 0.05) was used to explore associations between each criterion variables (verbal memory measures) and predictor variables (sociodemographic and disease characteristics: WM and language), and between predictor variables in each patient group. Kendall or point biserial correlation analyses were conducted between scale and ordinal, and between scale and categorical variables.

In the second step, hierarchical linear regressions were conducted in each patient group, with the verbal memory and learning variables as the dependent variable. The stepwise method was used, and standardized beta (β) values were reported. The hierarchical regression analysis results consist of model comparisons and a model interpretation based on an alpha of 0.05. Each step in the hierarchical regression was compared to the previous step using *F*-tests. The coefficients of the model in the final step were interpreted. For step 1, the relevant sociodemographic and disease characteristics were entered as predictor variables into the null model. Only sociodemographic and disease characteristics variables showing significant correlations with study variable (*r* > 0.30, *p* < 0.05) were first introduced in the regression models. Furthermore, depending on the specific research question for each criterion variable, either WM or language variables were separately added into the model in step 2 and step 3, respectively. At each step, variance inflation factors (VIFs) were calculated to detect the presence of multicollinearity between predictors. Then, variables generating the highest VIF values were subsequently excluded until all VIF values were <2. The following set of sociodemographic and disease-characteristic variables emerged as the most robust predictors in patient groups: education (years), age (at assessment), age at onset, epilepsy duration, frequency of seizures per year, and number of AEDs. However, age at onset was eliminated from almost all regression models as generating very high multicollinearity (VIF > 10). Where only one significant predictor was identified, linear regression was performed.

A Baron and Kenny mediation analysis (50) was conducted in each patient group to assess if WM mediates the relationship between the number of AED and short-term verbal memory measures: list-learning trial 1, learning capacity, and story memory. To determine whether a mediating relationship was supported by the data, three regressions were conducted. For mediation to be supported, four items must be met: (1) the independent variable (AED number) must be related the dependent variable (short-term verbal memory measure), (2) the independent variable (AED number) must be related to the mediator variable (WM), (3) the mediator must be related to the dependent variable but in the presence of the independent variable, and (4) the independent variable should no longer be a significant predictor of the dependent variable in the presence of the mediator variable (41). Only mediations supported by the data were further described in the results section.

Based on findings from the previous two steps, in the third step, ANCOVA was used to compare again the performance of groups at verbal memory while controlling for the relevant predictors.

Effect sizes were reported based on Cohen's standards (51), where coefficients between 0.10 and 0.29 represent a small effect size, coefficients between 0.30 and 0.49 represent a moderate effect size, and coefficients above 0.50 indicate a large effect size. There were no missing data. Correlation and regression analyses were performed with Intellectus Statistics™ (52). All other analyses were performed with SPSS Statistics for Windows, Version 23.0 (IBM Corp., Armonk, NY, USA) (53). *Post*-*hoc* effect size and achieved power for regression analyses were computed using G^*^Power 3.1.9.7 tool (54).

## Results

### Verbal Learning and Memory Performance in Patient Groups

Except for phonemic fluency, *F*(2, 107) = 2.297, *p* = 0.106, ηp^2^ = 0.04, participant groups performance analysis (Table 1) revealed significant differences between groups at all neuropsychological tests: list-learning capacity, χ^2^(2) = 13.73, *p* = 0.001; list-learning trial 1, χ^2^(2) = 11.02, *p* = 0.004; list-learning trial 5, χ^2^(2) = 14.94, *p* < 0.001; list-learning delayed-recall, χ^2^(2) = 19.03, *p* < 0.001; story memory, χ^2^(2) = 28.34, *p* < 0.001; picture naming, χ^2^(2) = 23.64, *p* < 0.001; semantic fluency, *F*(2, 107) = 7.90, *p* < 0.001, ηp^2^ = 0.13; and WM, χ^2^(2) = 19.12, *p* < 0.001. Pairwise *post*-*hoc* comparisons revealed that, compared to controls, both patient groups had impaired WM (LTLE: *p* = 0.002; RTLE: *p* < 0.001), but only the LTLE group was impaired at verbal memory and language measures: learning capacity (*p* = 0.001), trial 1 (*p* = 0.003), trial 5 (*p* < 0.001), delayed-recall (*p* < 0.001), story memory (*p* < 0.001), picture naming (*p* < 0.001), and semantic fluency (*p* < 0.001), whereas the RTLE group showed only a story memory impairment (*p* = 0.002). Compared to the RTLE group, LTLE showed a lower performance only at picture naming test (*p* = 0.017).

### Correlations Between Study Variables

Detailed correlations analysis results are included in Supplementary Material 1 for all participant groups, including 95% CI. In LTLE, WM showed a strong correlation with list-learning trial 1 (*r*_s_ = 0.53, *p* = 0.002), and moderate correlations with list-learning capacity (*r*_s_ = 0.46, *p* < 0.001) and trial 5 (*r*_s_ = 0.39, *p* = 0.009). Language variables showed moderate correlations with all verbal memory measures (*r*_s_ between 0.31 and 0.39, *p* < 0.05). In RTLE, WM showed a strong correlation only with story memory (*r*_s_ = 0.66, *p* < 0.001), whereas language (mainly picture naming) showed moderate and strong correlations with all verbal memory measures (*r*_s_ between 0.39 and 0.51, *p* < 0.05). We found no correlation between WM and language measures in either patient groups. Potential predictors showed no correlation with demographic and disease-characteristics in either patient groups, except a strong correlation between verbal fluency measures and education years in the RTLE group (r_s_ between 0.54 and 0.56, *p* < 0.01), and a moderate correlation between WM and AED number in the LTLE group (*r*_s_ = −0.37, *p* = 0.014). The AED number showed moderate correlations with all verbal learning and memory measures only in the LTLE group (*r*_s_ between −0.33 and −0.41, *p* < 0.05). IQ level (three levels: inferior, average, and superior) showed small-to-moderate correlations with the list-learning measures not only in LTLE (*r*_k_ between 0.33 and 0.45, *p* < 0.05) but also having a strong correlation with education years (*r*_k_ = 0.64, *p* < 0.001). Education had moderate correlations with list-learning capacity and trial 5 only in LTLE, and with story memory in RTLE, whereas age moderately correlated with list-learning delayed-recall in both groups, and with list-learning capacity in LTLE (*r*_s_ between 0.34 and 0.48, *p* < 0.05). We found no significant correlations between sex, history of traumatic brain injury (TBI) or focal to bilateral tonic-clonic seizures (FBTCS), and verbal memory variables in any patient group, except a moderate correlation between history of febrile convulsion (HFC) and list-learning capacity, trial 1 and delayed-recall in LTLE (*r*_pb_ between 0.31 and 0.41, *p* < 0.05).

In healthy control group, both WM and language showed moderate and strong correlations with verbal memory variables (*r*_s_ between 0.32 and 0.80, *p* < 0.001). In addition, WM had strong correlations with all language measures (*r*_s_ between 0.61 and 0.67, *p* < 0.001). Education had strong positive correlations with all verbal memory measures (*r*_s_ between 0.68 and 0.79, *p* < 0.001), whereas age showed moderate correlations only with list-learning measures (*r*_s_ between 0.31 and 0.49, *p* < 0.05). The IQ level strongly correlated with all verbal memory measures (*r*_k_ between 0.61 and 0.72, *p* < 0.001), and with education (*r*_k_ = 0.77, *p* < 0.001).

### Predictors of the Verbal Learning and Memory in Participant Groups

The results of regression analyses for variables predicting verbal learning and memory in each participant group are described in Supplementary Material 2. Tables 2–4 summarize the model comparisons for variables predicting verbal learning and memory in each participant groups. Each step was compared to the previous model in the hierarchical regression analysis. After controlling for relevant demographic and disease-characteristic variables, we found an opposite pattern of predictors in patient groups. In LTLE, we found WM as the sole predictor for learning capacity, explaining an additional 10% of the variation (*f*^2^ = 1.23, achieved power 0.99), and for trial 1 performance (additional 17%; *f*^2^ = 0.51, achieved power 0.98). Semantic fluency was the unique predictor for delayed-recall, explaining an additional 9% of the variation (*f*^2^ = 0.24, achieved power 0.80). No significant predictors were identified for trial 5 and story memory. In contrast, in RTLE, we found picture naming as the unique predictor for all list-learning measures (except for trial 1), explaining an additional 40% of the learning capacity variation (*f*^2^ = 0.33, achieved power 0.81), an additional 25% of the list-learning trial 5 variation (*f*^2^ = 0.35, achieved power 0.83), and an additional 23% of delayed-recall variation (*f*^2^ = 0.60, achieved power 0.92). WM was the unique predictor for story memory, explaining an additional 38% of the variation (*f*^2^ = 1.33, achieved power 0.99). No significant predictor was identified for list-learning trial 1.

**Table 2:** Model comparisons for variables predicting verbal learning and memory in LTLE.

**Variable**							
**(model)**	**Predictors**	***R^2^***	**df_mod_**	**df_res_**	***F***	***p***	**Δ*R*^2^**
**List-learning capacity**							
Step 1	education, age, epilepsy duration, frequency of seizures, AED number	0.42	4	39	6.96	<0.001	0.42
Step 2	WM added	0.52	1	38	8.13	0.007	0.10
Step 3	picture naming added	0.56	1	37	3.81	0.058	0.04
**List-learning T1**							
Step 1	epilepsy duration, AED number	0.22	2	41	5.69	0.007	0.22
Step 2	WM added	0.38	1	40	10.87	0.002	0.17
**List-learning T1**							
Step 1	education, AED number	0.29	2	41	8.19	0.001	0.29
Step 2	picture naming added	0.34	1	40	3.47	0.070	0.06
Step 3	WM added	0.36	1	39	0.97	0.330	0.02
**List-learning delayed recall**							
Step 1	age, AED number;	0.25	2	41	6.86	0.003	0.25
Step 2	semantic fluency added	0.34	1	40	5.45	0.025	0.09
**Story memory**							
Step 1	AED number	0.11	1	42	5.43	0.025	0.11
Step 2	semantic fluency added	0.19	1	41	3.74	0.060	0.07

*Each step was compared to the previous model in the hierarchical regression analysis. AED, antiepileptic drugs; LTLE, left drug-resistant temporal lobe epilepsy; WM, working memory*.

**Table 3:** Model comparisons for variables predicting verbal learning and memory in RTLE.

**Variable**							
**(model)**	**Predictors**	***R* ^2^**	**df_mod_**	**df_res_**	***F***	***p***	**Δ*R*^2^**
List-learning capacity^*^							
	picture naming	0.40	1	24	15.97	<0.001	-
List-learning T5^*^							
	picture naming	0.25	1	24	7.87	0.010	-
List-learning delayed-recall^**^							
Step 1	age	0.10	1	24	2.60	0.120	0.10
Step 2	picture naming added	0.33	1	23	7.78	0.010	0.23
Story memory^**^							
Step 1	education, age at onset	0.21	2	23	3.12	0.063	0.21
Step 2	WM added	0.59	1	22	20.47	<0.001	0.38
Step 3	semantic fluency added	0.59	1	21	0.03	0.862	0.00

* *Linear regression;*

** *Each step was compared to the previous model in the hierarchical regression analysis; WM, working memory; RTLE, right drug-resistant temporal lobe epilepsy*.

**Table 4:** Model comparisons for variables predicting verbal learning and memory in healthy controls.

**Variable**	**Predictors**	***R* ^2^**	**df_mod_**	**df_res_**	***F***	***p***	**Δ*R*^2^**
**List-learning capacity**							
Step 1	age	0.29	1	38	15.45	<0.001	0.29
Step 2	WM	0.52	1	37	17.87	<0.001	0.23
Step 3	picture naming and phonemic fluency	0.62	2	35	4.48	0.019	0.10
**List-learning T1**							
Step 1	age	0.13	1	38	5.91	0.020	0.13
Step 2	WM	0.37	1	37	13.81	<0.001	0.24
Step 3	picture naming and phonemic fluency	0.46	2	35	2.94	0.066	0.09
**List-learning T5**							
Step 1	age	0.15	1	38	6.66	0.014	0.15
Step 2	picture naming and phonemic fluency	0.38	2	36	6.78	0.003	0.23
Step 3	WM	0.46	1	35	4.83	0.035	0.07
**List-learning delayed-recall**							
Step 1	age	0.21	1	38	10.39	0.003	0.21
Step 2	picture naming and phonemic fluency	0.48	2	36	9.41	<0.001	0.27
Step 3	WM	0.56	1	35	6.17	0.018	0.08
**Story memory**							
Step 1	age	0.10	1	38	4.29	0.045	0.10
Step 2	WM	0.52	1	37	31.94	<0.001	0.42
Step 3	picture naming and phonemic fluency	0.60	2	35	3.81	0.032	0.09

*Each step was compared to the previous model in the hierarchical regression analysis*.

We additionally explored the relationships between WM, language, and verbal memory in the healthy control group. Education and age were introduced in the regression analyses in the first step. However, education was removed from all regression models as generating very high multicollinearity (VIF > 10). WM brought significant additional variation in all verbal memory measures: learning capacity (23%), trial 1 (24%), trial 5 (7%), delayed-recall (8%), and story memory (42%). From language measures, phonemic fluency added a significant amount of variation for all verbal memory measures except for trial 1: list-learning capacity (additional 10%), trial 5 (additional 23%), delayed-recall (additional 27%), and story memory (additional 7%). The regression model for list-learning trial 1 indicates that language did not account for a significant amount of additional variation (*p* = 0.066).

Table 5 summarizes the contribution of predictor variables to the verbal learning and memory performance (final model interpretation) in each of the participant groups (patients and healthy controls) after controlling for relevant disease characteristics and/or sociodemographic variables.

**Table 5:** Predictor variables for verbal learning and memory measures in participant groups, after controlling for disease characteristics and/or sociodemographic relevant variables (final model interpretation).

		**Healthy Controls**				**LTLE**				**RTLE**		
**Verbal memory variable**	**Predictor variable(s)**	**β value**	***t*-value**	***p*-value**	**Predictor**	**β value**	***t*-value**	***p*-value**	**Predictor**	**β value**	***t*-value**	***p*-value**
					**variable(s)**				**variable(s)**			
List-learning capacity	WM Phonemic Fluency	0.34 0.38	2.44 2.81	0.020 0.008	WM	0.30	2.42	0.020	Picture naming	0.63	4.00	<0.001
List-learning Trial 1	WM Phonemic Fluency	0.42 0.38	2.54 2.41	0.016 0.021	WM	0.44	3.30	0.002	-	-	-	-
List-learning Trial 5	Phonemic Fluency WM	0.44 0.37	2.78 2.20	0.009 0.035	-	-	-	-	Picture naming	0.50	2.81	0.010
List-learning Delayed-recall	Phonemic Fluency WM	0.44 0.37	3.06 2.48	0.004 0.018	Semantic Fluency	0.31	2.33	0.025	Picture naming	0.48	2.79	0.010
Story memory	WM Phonemic Fluency	0.62 0.37	4.30 2.73	<0.001 0.010	-	-	-	-	WM	0.61	3.97	<0.001

*LTLE, left drug-resistant temporal lobe epilepsy; RTLE, right drug-resistant temporal lobe epilepsy; WM, working memory; β-value, standardized beta coefficient*.

For the last step of our analysis, participant groups were again compared for verbal memory measures, taking relevant predictors as covariates. When controlling for WM, the results of the ANCOVA were not significant for list-learning trial 1, *F*(2, 106) = 2.709, *p* = 0.071, η^2^ = 0.05, and significant for story memory, *F*(2, 106) = 10.527, *p* < 0.001, η^2^ = 0.17; the mean of story memory for controls (M = 13.93, SD = 3.54) was significantly larger than for LTLE (M = 10.37, SD = 3.41), *p* < 0.001, but similar with RTLE (*p* > 0.05). WM confounds the performance at the short-term verbal memory measures: story memory in RTLE, and list-learning trial 1 in LTLE.

When controlling for age at assessment, education, picture naming, and WM, the results were not significant for learning capacity, *F*(2, 103) = 2.128, *p* = 0.124, η^2^ = 0.04. However, when each of the predictors were controlled separately, we found that only picture naming confounds the performance at the list-learning capacity measure, *F*(2, 106) = 2.157, *p* = 0.121, η^2^ = 0.04. When controlling for education years and picture naming, the results were significant for list-learning trial 5, *F*(2, 105) = 4.834, *p* = 0.01, η^2^ = 0.08; the mean of trial 5 for control (M = 13.08, SD = 1.71) was significantly larger than for LTLE (M = 11.86, SD = 1.73), *p* = 0.007. When controlling for age at assessment, picture naming, and semantic fluency, the results of the ANCOVA were significant for list-learning delayed-recall, *F*(2, 104) = 4.419, *p* = 0.014, η^2^ = 0.08; the mean of list-learning delayed-recall for control (M = 11.35, SD = 2.32) was significantly larger than for LTLE (M = 9.81, SD = 2.31), *p* = 0.014.

### WM as a Mediator Between AED Number and Short-Term Memory Measures

Our data analyses revealed that the mediating relationship was supported only for list-learning capacity and list-learning trial 1 in LTLE. No mediation relationships were identified in RTLE between WM, AED number, and any of the short-term verbal memory measures.

#### List-Learning Trial 1 (LTLE)

In step 1 of the mediation model, the regression of AED number on list-learning, ignoring the mediator, was significant, β = −0.95, *t* = −2.80, *p* < 0.01. Step 2 showed that the regression of the AED number on WM was also significant, β = −1.76, *t* = −2.57, *p* < 0.05. Step 3 showed that the effect of WM on the list-learning, controlling for AED number, was significant, β = 0.22, *t* = 3.16, *p* < 0.01, whereas in the presence of the mediator, the AED number was not a significant predictor of list-learning, β = −0.56, *t* = −1.71, *p* = 0.09. Complete mediation is supported.

#### List-Learning Capacity (LTLE)

In step 1 of the mediation model, the regression of AED number on list-learning capacity, ignoring the mediator, was significant, β = −5.32, *t* = −3.12, *p* < 0.01. In step 2, the regression of the AED number on WM was also significant, β = −1.76, *t* = −2.57, *p* < 0.05. Step 3 showed that effect of WM on the list-learning capacity, controlling for AED number, was significant, β = 0.89, *t* = 2.45, *p* < 0.05, and AED number was still a significant predictor of list-learning capacity, whereas in the presence of the mediator WM (β = −3.75, *t* = −2.16, *p* < 0.05). Partial mediation is supported.

## Discussion

We conducted this study intending to understand the individual contributions of WM and language in the verbal learning and memory performance of patients with unilateral drTLE, beyond the influence of demographic and disease characteristics. Additionally, we explored the mediating role of the WM on the relationship between the number of AED and short-term verbal memory.

Similar to previous findings (10–12), we found patients with drTLE impaired at WM irrespective of their seizure onset lateralization. In line with the current neuropsychological literature, patients with LTLE were impaired at semantic-related language tasks (picture naming and semantic fluency) and at all verbal-learning measures when compared to healthy controls while showing lower naming abilities compared to the patients with RTLE. Compared to controls, both patient groups were impaired at the story memory test, which is similar to previous findings (17, 55–59).

As we hypothesized, in the two groups, the short-term verbal memory measures were predicted by WM, but differently: the total learning capacity and the first trial of the list-learning task in the LTLE group, and the story memory in the RTLE group. The final comparison with healthy controls revealed that WM confounds the performance at these measures in both groups. The last list-learning trial, a measure of learning ability, was predicted exclusively by language in the RTLE group, while showing no relevant predictor in the LTLE group. In RTLE group, picture naming was the unique predictor for most list-learning measures, whereas in the LTLE group, semantic fluency showed a significant but small contribution on the list-learning delayed-recall measure. Finally, after controlling for relevant predictors, patients with LTLE performed worse than controls only at list-learning last trial, delayed-recall, and story memory tests. In addition, in line with previous research (26), we found that naming abilities confounds the performance at the list-learning capacity measure in the LTLE group. Thus, in line with the literature, only patients with LTLE have verbal memory consolidation and retrieval deficits and a semantic-related impairment.

Many neuropsychological studies addressing the verbal memory performance in drTLE did not consider the contribution of other cognitive processes, like WM and language, on the verbal memory performance. We found both WM and language as significant predictors of various verbal memory components, with WM confounding the performance at the short-term memory measures. Our analyses show that the magnitude of the effect of WM on verbal learning seems to decrease over the list-learning trials, showing no effect on the last trial and the delayed-recall measures, but only in the LTLE group. Conversely, in the RTLE group, WM was associated only with story memory but not with any of the list-learning measures, most probably due to the protective and compensatory role played by the better language abilities in this group. In healthy controls, we found that WM contributes, in addition to language, to all verbal memory measures, whereas being the strongest predictor for the short-term memory measures (list-learning trial 1 and story memory—immediate recall). Thus, our findings support the multi-store memory model (60), where various memory components dynamically interact in the learning process, and control processes such as “rehearsal” play an essential role in the transfer of information from the short-term to the long-term memory (61).

In addition, the current study adds to the findings that semantic function, characterized by deficits in semantic information retrieval (naming, semantic verbal fluency) and impaired performance at semantic verbal memory task (i.e., story memory), is altered in language-dominant patients with drTLE (62). The interdependence between verbal memory and language networks within the left temporal lobe is supported by many neuroimaging studies (63).

Some essential observations can be drawn so far. Story memory is impaired in patients with LTLE independent of their WM abilities. In addition, the list-learning last trial can be considered a better verbal learning ability indicator than the overall learning capacity measure (i.e., the sum of the five trials), proving to be impaired in patients with LTLE independent of language and WM.

As previously suggested (33), our findings showed that WM mediates the interaction between the AED number and the short-term verbal learning measures (i.e., the list-learning first trial and the total learning capacity measure), but this effect was seen only in patients with LTLE. AED was shown to have a differential impact on verbal memory (64), particularly in patients with LTLE (65), mainly when administrated in high doses and polytherapy (66–69).

We found that none of the verbal memory measures differentiated LTLE from RTLE even after controlling for relevant predictors, consistent with other studies (17, 22, 55, 58, 70). However, the story memory task used in our study required immediate recall after only one presentation of the material; therefore, it is representative of the short-term verbal memory. The performance at memory for verbal passages in a learning paradigm showed a significant difference between patients with LTLE and RTLE (71). As such, it would be more informative to understand the contribution of WM and language on various stages of a story memory learning paradigm, including delayed-recall.

We observed that an opposite pattern of contributors emerged in patient groups. Several reasons might explain this discrepancy. First, the lexical-semantic retrieval adequacy (mainly naming abilities) may be the main reason for this discrepancy, playing a protective and compensatory role for patients with RTLE and overcoming their deficient WM.

Second, the Letter-Number Sequencing task used to measure WM in this study might measure the verbal buffer, a domain-specific “slave system” (43), rather than measuring more complex executive functions (72). Strong neuroimaging evidence connects verbal WM with the left hemisphere (73). In particular, the neuroanatomical basis of the phonological loop is mainly in the left hemisphere, reflecting a strong connection with language networks (74). The verbal learning and memory tests used in this study were delivered to participants in the auditory presentation modality, which presumably relies on the phonological loop (75).

Third, most probable, the language tasks used in our study did not capture the complexity of the language–memory relationship involved by the story memory test. Traditionally, language impairment is well recognized in drTLE for verbal fluency (76) and naming abilities (77), whereas other important language expressive functions, like spontaneous speech and discourse abilities, are much less addressed by the literature (78). Although basic language functions are generally considered unaffected in patients with TLE, the narrative discourse seems to be affected (79), particularly concerning discourse and high-level language abilities (80), probably associated with low WM capacity (81). Patients with early-onset TLE show mild discourse production impairments not associated with other language measures, but correlating with WM (82). In healthy subjects, WM emerged as a robust predictor for high-level language abilities (83). Maintaining verbal information in the verbal WM system directly depends on the long-term representations and processes used in language comprehension and production (84, 85). Retelling a story must involve language production processes as it naturally involves the maintenance and ordering of linguistic information during spoken recall (86). LTLE is particularly associated with macro linguistic disturbances affecting discourse production (87) and spontaneous speech (88). Postoperatively, patients with LTLE were found to be impaired at the immediate recall of stories when compared to those with RTLE, although preoperatively they had similar impaired performance, and this result was not related to the WM capacity, being most probably due to the language network disturbance following left anterior temporal lobe resection (89). Similarly, Joo et al. (2005) (90) found correlations between the extent of left temporal lobe surgery and performance at the story memory test. Thus, the adequacy of the language system of patient, both receptive and expressive, critically influences the interictal verbal learning and memory and must be included in interpreting the memory tests results (78).

### Clinical Implications

Working memory is impaired in drTLE and predicts verbal learning and memory deficit, in addition to the language. Clinicians should consider this overlap when interpreting poor performance in verbal learning and memory assessment by including a relevant WM task and examining the learning performance over the five trials of a list-learning test. This is particularly important when naming abilities or other language functions are within the normal range. Our findings suggest that the last list-learning trial is more informative for the verbal learning integrity of the language-dominant hemisphere than the traditional learning capacity measure (the sum of the five trials). Instead, the performance at the first trial showed the highest sensitivity to a WM impairment. Additionally, an impairment at the story memory test in the absence of a WM deficit may suggest that the functional integrity of the language-dominant hemisphere is affected. Concurrently, a deficit at the story memory test should be interpreted with caution when WM is impaired.

### Limitations

Our patient group included various pathologies; therefore, we could not disentangle the effect of mesial or lateral temporal lobe epilepsy on study variables. In addition, besides hand-dominance, we did not use another modality to determine the language lateralization; therefore, we can only assume that the majority of our patients have the left hemisphere as language-dominant (91, 92). Although the sample sizes of patients are small, particularly for RTLE group, the *post*-*hoc* effect sizes and the achieved power for regression analyses showed significant results in each group. In addition to the AED number, further research should explore the mediating role of WM on the relationship between specific drugs (i.e., type, dosage, and combination) and verbal memory performance in drTLE.

## Data Availability Statement

The datasets presented in this study can be found in online repositories. The name of the repository and accession number can be found below: Mendeley Data, https://data.mendeley.com, doi: 10.17632/hhwh69wy6n.1.

## Ethics Statement

The studies involving human participants were reviewed and approved by Ethics Board of the University of Bucharest, Romania (notice no. 329/11.12.2019). The participants provided their written informed consent to participate in this study.

## Author Contributions

MB and CI contributed to the conception, the design of the study, and its implementation. MB wrote the first draft of the manuscript. CI wrote sections of the manuscript. CI and EA reviewed the manuscript. All authors read and approved the submitted version.

## Conflict of Interest

The authors declare that the research was conducted in the absence of any commercial or financial relationships that could be construed as a potential conflict of interest.

## Publisher's Note

All claims expressed in this article are solely those of the authors and do not necessarily represent those of their affiliated organizations, or those of the publisher, the editors and the reviewers. Any product that may be evaluated in this article, or claim that may be made by its manufacturer, is not guaranteed or endorsed by the publisher.
