# Supplementary material for: Working Memory and Language Contribution to Verbal Learning and Memory in Drug-Resistant Unilateral Focal Temporal Lobe Epilepsy
Source: Front Neurol. 2021 Dec 8;12:780086. doi: 10.3389/fneur.2021.780086 (PMC8692669; doi:10.3389/fneur.2021.780086)
Supplement: Supplementary file 1 [file Table_1.DOCX]

***Supplementary Material 1***

**Correlation Analysis between study variables in each participants’ group**

**Spearman Correlation Analysis**

***Introduction***

A Spearman correlation analysis was conducted among study variables in each group. Cohen's standard was used to evaluate the strength of the relationships, where coefficients between .10 and .29 represent a small effect size, coefficients between .30 and .49 represent a moderate effect size, and coefficients above .50 indicate a large effect size (Cohen, 1988). The result of the correlations was examined based on an alpha value of 0.05. Tables 1-6 present the results of the correlations.

**Table 1**

*Spearman Correlation Results Among study verbal learning and memory variables and potential predictor variables (LTLE)*

| Combination | *r*_s_ | 95% CI | *p* |
| --- | --- | --- | --- |
| List-learning capacity-WM | 0.46 | [0.19, 0.67] | .002 |
| List-learning capacity-Picture naming | 0.32 | [0.03, 0.56] | .034 |
| List-learning capacity-Semantic fluency | 0.37 | [0.08, 0.60] | .014 |
| List-learning capacity-Phonemic fluency | 0.24 | [-0.06, 0.50] | .118 |
| List-learning T1-WM | 0.53 | [0.27, 0.71] | < .001 |
| List-learning T1-Picture naming | 0.25 | [-0.05, 0.51] | .106 |
| List-learning T1-Semantic fluency | 0.28 | [-0.02, 0.53] | .065 |
| List-learning T1-Phonemic fluency | 0.13 | [-0.18, 0.41] | .409 |
| List-learning T5-WM | 0.39 | [0.10, 0.61] | .009 |
| List-learning T5-Picture naming | 0.37 | [0.08, 0.60] | .013 |
| List-learning T5-Semantic fluency | 0.29 | [-0.01, 0.54] | .056 |
| List-learning T5-Phonemic fluency | 0.39 | [0.11, 0.62] | .008 |
| List-learning delayed-WM | 0.19 | [-0.11, 0.46] | .206 |
| List-learning delayed-Picture naming | 0.24 | [-0.06, 0.50] | .119 |
| List-learning delayed-Semantic fluency | 0.31 | [0.01, 0.55] | .042 |
| List-learning delayed-Phonemic fluency | 0.18 | [-0.12, 0.46] | .230 |
| Story Memory-WM | 0.21 | [-0.10, 0.47] | .180 |
| Story Memory-Picture naming | 0.22 | [-0.08, 0.49] | .145 |
| Story Memory-Semantic fluency | 0.36 | [0.07, 0.59] | .016 |
| Story Memory-Phonemic fluency | -0.09 | [-0.37, 0.22] | .579 |

*Note. n = 44. LTLE = patients with left drug-resistant temporal lobe epilepsy; WM = working memory*

**Table 2**

*Spearman Correlation Results Among study verbal learning and memory variables and potential predictor variables (RTLE)*

| Combination | *r*_s_ | 95% CI | *p* |
| --- | --- | --- | --- |
| List-learning capacity-WM | 0.17 | [-0.24, 0.52] | .414 |
| List-learning capacity-Picture naming | 0.50 | [0.14, 0.74] | .009 |
| List-learning capacity-Semantic fluency | 0.39 | [0.00, 0.68] | .049 |
| List-learning capacity-Phonemic fluency | 0.31 | [-0.09, 0.62] | .128 |
| List-learning T1-WM | -0.02 | [-0.41, 0.37] | .909 |
| List-learning T1-Picture naming | 0.25 | [-0.15, 0.58] | .209 |
| List-learning T1-Semantic fluency | 0.20 | [-0.20, 0.55] | .324 |
| List-learning T1-Phonemic fluency | 0.22 | [-0.19, 0.56] | .285 |
| List-learning T5-WM | 0.14 | [-0.26, 0.50] | .490 |
| List-learning T5-Picture naming | 0.51 | [0.15, 0.75] | .008 |
| List-learning T5-Semantic fluency | 0.29 | [-0.11, 0.61] | .150 |
| List-learning T5-Phonemic fluency | 0.25 | [-0.15, 0.58] | .215 |
| List-learning delayed-WM | 0.12 | [-0.28, 0.48] | .569 |
| List-learning delayed-Picture naming | 0.47 | [0.10, 0.72] | .016 |
| List-learning delayed-Semantic fluency | 0.37 | [-0.03, 0.66] | .066 |
| List-learning delayed-Phonemic fluency | 0.24 | [-0.17, 0.57] | .247 |
| Story Memory-WM | 0.66 | [0.37, 0.83] | < .001 |
| Story Memory-Picture naming | 0.22 | [-0.18, 0.56] | .275 |
| Story Memory-Semantic fluency | 0.39 | [0.00, 0.68] | .048 |
| Story Memory-Phonemic fluency | 0.16 | [-0.24, 0.52] | .429 |

*Note. n = 26. RTLE = patients with right drug-resistant temporal lobe epilepsy; WM = working memory*

**Table 3**

*Spearman Correlation Results Among verbal learning and memory variables and socio-demographic and disease characteristics (LTLE)*

| Combination | *r*_s_ | 95% CI | *p* |
| --- | --- | --- | --- |
| List-learning capacity-Education | 0.34 | [0.05, 0.58] | .024 |
| List-learning capacity-Age | -0.39 | [-0.62, -0.11] | .008 |
| List-learning capacity-Age at onset | -0.07 | [-0.36, 0.23] | .661 |
| List-learning capacity-Epilepsy duration | -0.35 | [-0.59, -0.06] | .020 |
| List-learning capacity-Frequency of seizures | -0.11 | [-0.39, 0.19] | .477 |
| List-learning capacity-AED number | -0.41 | [-0.63, -0.13] | .005 |
| List-learning T1-Education | 0.17 | [-0.14, 0.44] | .280 |
| List-learning T1-Age | -0.14 | [-0.42, 0.16] | .355 |
| List-learning T1-Age at onset | 0.17 | [-0.13, 0.44] | .268 |
| List-learning T1-Epilepsy duration | -0.34 | [-0.58, -0.05] | .022 |
| List-learning T1-Frequency of seizures | -0.00 | [-0.30, 0.29] | .979 |
| List-learning T1-AED number | -0.39 | [-0.62, -0.11] | .008 |
| List-learning T5-Education | 0.48 | [0.22, 0.68] | < .001 |
| List-learning T5-Age | -0.22 | [-0.48, 0.08] | .153 |
| List-learning T5-Age at onset | -0.00 | [-0.30, 0.30] | .992 |
| List-learning T5-Epilepsy duration | -0.24 | [-0.50, 0.07] | .124 |
| List-learning T5-Frequency of seizures | -0.07 | [-0.36, 0.23] | .657 |
| List-learning T5-AED number | -0.35 | [-0.59, -0.06] | .019 |
| List-learning delayed-Education | 0.25 | [-0.05, 0.51] | .099 |
| List-learning delayed-Age | -0.35 | [-0.59, -0.06] | .019 |
| List-learning delayed-Age at onset | -0.06 | [-0.35, 0.24] | .683 |
| List-learning delayed-Epilepsy duration | -0.29 | [-0.54, 0.00] | .053 |
| List-learning delayed-Frequency of seizures | -0.13 | [-0.41, 0.17] | .399 |
| List-learning delayed-AED number | -0.34 | [-0.58, -0.04] | .026 |
| Story Memory-Education | 0.26 | [-0.04, 0.52] | .086 |
| Story Memory-Age | -0.18 | [-0.45, 0.13] | .250 |
| Story Memory-Age at onset | 0.05 | [-0.25, 0.34] | .742 |
| Story Memory-Epilepsy duration | -0.22 | [-0.49, 0.08] | .148 |
| Story Memory-Frequency of seizures | -0.14 | [-0.42, 0.17] | .376 |
| Story Memory-AED number | -0.33 | [-0.57, -0.03] | .030 |

*Note. n = 44. LTLE = patients with left drug-resistant temporal lobe epilepsy*

**Table 4**

*Spearman Correlation Results Among verbal learning and memory variables and socio-demographic and disease characteristics (LTLE)*

| Combination | *r*_s_ | 95% CI | *p* |
| --- | --- | --- | --- |
| List-learning capacity-Education | 0.13 | [-0.27, 0.49] | .528 |
| List-learning capacity-Age | -0.34 | [-0.64, 0.05] | .089 |
| List-learning capacity-Age at onset | -0.15 | [-0.51, 0.25] | .470 |
| List-learning capacity-Epilepsy duration | -0.12 | [-0.48, 0.28] | .571 |
| List-learning capacity-Frequency of seizures | 0.09 | [-0.31, 0.46] | .670 |
| List-learning capacity-AED number | 0.10 | [-0.30, 0.47] | .633 |
| List-learning T1-Education | 0.01 | [-0.38, 0.40] | .951 |
| List-learning T1-Age | -0.23 | [-0.56, 0.18] | .266 |
| List-learning T1-Age at onset | -0.01 | [-0.40, 0.38] | .963 |
| List-learning T1-Epilepsy duration | -0.17 | [-0.52, 0.23] | .402 |
| List-learning T1-Frequency of seizures | 0.31 | [-0.09, 0.62] | .125 |
| List-learning T1-AED number | -0.21 | [-0.55, 0.19] | .298 |
| List-learning T5-Education | -0.04 | [-0.42, 0.35] | .849 |
| List-learning T5-Age | -0.33 | [-0.64, 0.06] | .095 |
| List-learning T5-Age at onset | -0.37 | [-0.66, 0.02] | .065 |
| List-learning T5-Epilepsy duration | 0.06 | [-0.33, 0.44] | .756 |
| List-learning T5-Frequency of seizures | 0.11 | [-0.29, 0.47] | .607 |
| List-learning T5-AED number | 0.23 | [-0.18, 0.56] | .264 |
| List-learning delayed-Education | 0.11 | [-0.29, 0.48] | .581 |
| List-learning delayed-Age | -0.39 | [-0.68, -0.01] | .047 |
| List-learning delayed-Age at onset | -0.38 | [-0.67, 0.01] | .058 |
| List-learning delayed-Epilepsy duration | 0.10 | [-0.30, 0.47] | .621 |
| List-learning delayed-Frequency of seizures | 0.14 | [-0.26, 0.50] | .506 |
| List-learning delayed-AED number | 0.35 | [-0.04, 0.65] | .079 |
| Story Memory-Education | 0.48 | [0.11, 0.73] | .014 |
| Story Memory-Age | 0.18 | [-0.22, 0.53] | .378 |
| Story Memory-Age at onset | 0.45 | [0.08, 0.71] | .020 |
| Story Memory-Epilepsy duration | -0.27 | [-0.60, 0.13] | .180 |
| Story Memory-Frequency of seizures | -0.09 | [-0.46, 0.30] | .650 |
| Story Memory-AED number | -0.09 | [-0.46, 0.31] | .667 |

*Note. n = 26. RTLE = patients with right drug-resistant temporal lobe epilepsy*

**Table 5**

*Spearman Correlation Results among predictors and socio-demographic and disease characteristics variables (LTLE)*

| Combination | *r*_s_ | 95% CI | *p* |
| --- | --- | --- | --- |
| WM-Picture naming | 0.24 | [-0.06, 0.50] | .118 |
| WM-Phonemic fluency | 0.26 | [-0.04, 0.52] | .084 |
| WM-Semantic fluency | 0.25 | [-0.05, 0.51] | .103 |
| WM-Education | 0.34 | [0.05, 0.58] | .023 |
| WM-Age | 0.09 | [-0.21, 0.38] | .552 |
| WM-Age at onset | 0.26 | [-0.04, 0.51] | .094 |
| WM-Epilepsy duration | -0.15 | [-0.42, 0.16] | .346 |
| WM-Frequency of seizures | -0.19 | [-0.46, 0.11] | .210 |
| WM-AED number | -0.37 | [-0.60, -0.08] | .014 |
| Picture naming-Phonemic fluency | 0.26 | [-0.03, 0.52] | .082 |
| Picture naming-Semantic fluency | 0.52 | [0.26, 0.71] | < .001 |
| Picture naming-Education | 0.24 | [-0.06, 0.50] | .111 |
| Picture naming-Age | 0.14 | [-0.17, 0.42] | .381 |
| Picture naming-Age at onset | 0.01 | [-0.29, 0.30] | .958 |
| Picture naming-Epilepsy duration | -0.05 | [-0.34, 0.25] | .756 |
| Picture naming-Frequency of seizures | -0.29 | [-0.54, 0.01] | .060 |
| Picture naming-AED number | -0.06 | [-0.35, 0.24] | .693 |
| Phonemic fluency-Semantic fluency | 0.33 | [0.03, 0.57] | .030 |
| Phonemic fluency-Education | 0.24 | [-0.06, 0.50] | .115 |
| Phonemic fluency-Age | 0.08 | [-0.22, 0.37] | .584 |
| Phonemic fluency-Age at onset | 0.06 | [-0.24, 0.35] | .686 |
| Phonemic fluency-Epilepsy duration | 0.11 | [-0.19, 0.40] | .470 |
| Phonemic fluency-Frequency of seizures | 0.03 | [-0.27, 0.33] | .838 |
| Phonemic fluency-AED number | -0.11 | [-0.40, 0.19] | .468 |
| Semantic fluency-Education | 0.17 | [-0.14, 0.44] | .280 |
| Semantic fluency-Age | -0.14 | [-0.42, 0.16] | .357 |
| Semantic fluency-Age at onset | -0.13 | [-0.41, 0.17] | .386 |
| Semantic fluency-Epilepsy duration | -0.07 | [-0.36, 0.24] | .671 |
| Semantic fluency-Frequency of seizures | -0.09 | [-0.38, 0.21] | .567 |
| Semantic fluency-AED number | -0.16 | [-0.44, 0.14] | .300 |
| Education-Age | 0.22 | [-0.08, 0.49] | .148 |
| Education-Age at onset | 0.24 | [-0.06, 0.50] | .114 |
| Education-Epilepsy duration | -0.03 | [-0.33, 0.27] | .826 |
| Education-Frequency of seizures | -0.24 | [-0.50, 0.06] | .114 |
| Education-AED number | -0.23 | [-0.50, 0.07] | .125 |
| Age-Age at onset | 0.57 | [0.33, 0.74] | < .001 |
| Age-Epilepsy duration | 0.32 | [0.03, 0.57] | .033 |
| Age-Frequency of seizures | -0.04 | [-0.33, 0.26] | .817 |
| Age-AED number | 0.22 | [-0.08, 0.49] | .149 |
| Age at onset-Epilepsy duration | -0.46 | [-0.67, -0.19] | .001 |
| Age at onset-Frequency of seizures | -0.28 | [-0.53, 0.02] | .067 |
| Age at onset-AED number | -0.24 | [-0.50, 0.06] | .110 |
| Epilepsy duration-Frequency of seizures | 0.38 | [0.10, 0.61] | .010 |
| Epilepsy duration-AED number | 0.51 | [0.25, 0.70] | < .001 |
| Frequency of seizures-AED number | 0.33 | [0.03, 0.57] | .030 |

*Note. n = 44. LTLE = patients with left temporal lobe epilepsy; WM = working memory*

**Table 6**. *Spearman Correlation Results among predictors and socio-demographic and disease characteristics variables (RTLE)*

| Combination | *r*_s_ | 95% CI | *p* |
| --- | --- | --- | --- |
| WM-Picture naming | 0.02 | [-0.37, 0.41] | .919 |
| WM-Phonemic fluency | -0.03 | [-0.41, 0.36] | .881 |
| WM-Semantic fluency | 0.37 | [-0.02, 0.66] | .065 |
| WM-Education | 0.17 | [-0.23, 0.53] | .395 |
| WM-Age | 0.17 | [-0.23, 0.53] | .396 |
| WM-Age at onset | 0.32 | [-0.07, 0.63] | .107 |
| WM-Epilepsy duration | -0.12 | [-0.49, 0.28] | .551 |
| WM-Frequency of seizures | -0.17 | [-0.52, 0.23] | .413 |
| WM-AED number | -0.02 | [-0.41, 0.37] | .905 |
| Picture naming-Phonemic fluency | 0.39 | [0.01, 0.68] | .047 |
| Picture naming-Semantic fluency | 0.33 | [-0.07, 0.63] | .104 |
| Picture naming-Education | 0.27 | [-0.13, 0.60] | .182 |
| Picture naming-Age | 0.01 | [-0.38, 0.39] | .968 |
| Picture naming-Age at onset | -0.14 | [-0.50, 0.26] | .485 |
| Picture naming-Epilepsy duration | -0.05 | [-0.43, 0.35] | .816 |
| Picture naming-Frequency of seizures | -0.06 | [-0.43, 0.34] | .786 |
| Picture naming-AED number | 0.00 | [-0.38, 0.39] | .988 |
| Phonemic fluency-Semantic fluency | 0.63 | [0.32, 0.82] | < .001 |
| Phonemic fluency-Education | 0.48 | [0.11, 0.73] | .013 |
| Phonemic fluency-Age | 0.12 | [-0.28, 0.49] | .549 |
| Phonemic fluency-Age at onset | -0.17 | [-0.52, 0.23] | .411 |
| Phonemic fluency-Epilepsy duration | 0.15 | [-0.25, 0.51] | .451 |
| Phonemic fluency-Frequency of seizures | 0.10 | [-0.30, 0.47] | .621 |
| Phonemic fluency-AED number | 0.01 | [-0.38, 0.39] | .974 |
| Semantic fluency-Education | 0.50 | [0.14, 0.74] | .010 |
| Semantic fluency-Age | 0.12 | [-0.28, 0.49] | .553 |
| Semantic fluency-Age at onset | 0.01 | [-0.38, 0.40] | .952 |
| Semantic fluency-Epilepsy duration | 0.19 | [-0.21, 0.54] | .344 |
| Semantic fluency-Frequency of seizures | -0.08 | [-0.46, 0.31] | .680 |
| Semantic fluency-AED number | -0.14 | [-0.50, 0.26] | .501 |
| Education-Age | 0.37 | [-0.02, 0.66] | .064 |
| Education-Age at onset | 0.32 | [-0.07, 0.63] | .109 |
| Education-Epilepsy duration | -0.05 | [-0.43, 0.35] | .816 |
| Education-Frequency of seizures | -0.17 | [-0.52, 0.23] | .405 |
| Education-AED number | -0.12 | [-0.48, 0.28] | .565 |
| Age-Age at onset | 0.39 | [-0.00, 0.67] | .051 |
| Age-Epilepsy duration | 0.16 | [-0.25, 0.51] | .449 |
| Age-Frequency of seizures | -0.22 | [-0.56, 0.19] | .286 |
| Age-AED number | -0.21 | [-0.55, 0.20] | .312 |
| Age at onset-Epilepsy duration | -0.70 | [-0.85, -0.43] | < .001 |
| Age at onset-Frequency of seizures | -0.46 | [-0.72, -0.09] | .018 |
| Age at onset-AED number | -0.44 | [-0.71, -0.06] | .025 |
| Epilepsy duration-Frequency of seizures | 0.38 | [-0.01, 0.67] | .057 |
| Epilepsy duration-AED number | 0.24 | [-0.17, 0.57] | .247 |
| Frequency of seizures-AED number | 0.34 | [-0.05, 0.64] | .089 |

*Note. n = 26; RTLE = patients with right temporal lobe epilepsy; WM = working memory*

**Table 7.** *Spearman Correlation Results Among study verbal learning and memory variables and potential predictor variables (Healthy Controls)*

| Combination | *r*_s_ | 95% CI | *p* |
| --- | --- | --- | --- |
| WM-Picture Naming | 0.67 | [0.45, 0.81] | < .001 |
| WM-Phonemic Fluency | 0.61 | [0.37, 0.78] | < .001 |
| WM-Semantic Fluency | 0.62 | [0.38, 0.78] | < .001 |
| WM-List-learning capacity | 0.65 | [0.43, 0.80] | < .001 |
| WM-List-learning T1 | 0.51 | [0.24, 0.71] | .001 |
| WM-List-learning T5 | 0.52 | [0.24, 0.71] | < .001 |
| WM-List-learning Delayed | 0.57 | [0.31, 0.75] | < .001 |
| WM-Story Memory | 0.66 | [0.43, 0.80] | < .001 |
| Picture Naming-Phonemic Fluency | 0.60 | [0.36, 0.77] | < .001 |
| Picture Naming-Semantic Fluency | 0.49 | [0.21, 0.70] | < .001 |
| Picture Naming-List-learning capacity | 0.56 | [0.30, 0.74] | < .001 |
| Picture Naming-List-learning T1 | 0.38 | [0.08, 0.62] | .016 |
| Picture Naming-List-learning T5 | 0.32 | [0.01, 0.57] | < .001 |
| Picture Naming-List-learning Delayed | 0.47 | [0.19, 0.68] | < .001 |
| Picture Naming-Story Memory | 0.39 | [0.09, 0.62] | < .001 |
| Phonemic Fluency-Semantic Fluency | 0.84 | [0.72, 0.91] | < .001 |
| Phonemic Fluency-List-learning capacity | 0.66 | [0.43, 0.80] | .046 |
| Phonemic Fluency-List-learning T1 | 0.56 | [0.30, 0.74] | < .001 |
| Phonemic Fluency-List-learning T5 | 0.57 | [0.32, 0.75] | < .001 |
| Phonemic Fluency-List-learning Delayed | 0.66 | [0.44, 0.81] | < .001 |
| Phonemic Fluency-Story Memory | 0.62 | [0.38, 0.78] | < .001 |
| Semantic Fluency-List-learning capacity | 0.80 | [0.66, 0.89] | < .001 |
| Semantic Fluency-List-learning T1 | 0.58 | [0.33, 0.76] | .002 |
| Semantic Fluency-List-learning T5 | 0.63 | [0.39, 0.79] | < .001 |
| Semantic Fluency-List-learning Delayed | 0.73 | [0.54, 0.85] | < .001 |
| Semantic Fluency-Story Memory | 0.64 | [0.42, 0.80] | < .001 |

*Note.* *n* = 40; *WM = working memory*

**Table 8.** *Spearman Correlation Results among study verbal learning and memory variables and socio-demographic characteristics (Healthy Controls)*

| Combination | *r*_s_ | 95% CI | *p* |
| --- | --- | --- | --- |
| Education-Age | -0.27 | [-0.54, 0.04] | .087 |
| Education-List-learning capacity | 0.77 | [0.60, 0.87] | < .001 |
| Education-List-learning T1 | 0.73 | [0.54, 0.85] | .001 |
| Education-List-learning T5 | 0.79 | [0.64, 0.89] | < .001 |
| Education-List-learning Delayed | 0.76 | [0.60, 0.87] | .055 |
| Education-Story Memory | 0.68 | [0.46, 0.82] | < .001 |
| Age-List-learning capacity | -0.49 | [-0.69, -0.21] | < .001 |
| Age-List-learning T1 | -0.31 | [-0.56, 0.01] | .037 |
| Age-List-learning T5 | -0.33 | [-0.58, -0.02] | < .001 |
| Age-List-learning Delayed | -0.43 | [-0.66, -0.14] | < .001 |
| Age-Story Memory | -0.28 | [-0.54, 0.03] | < .001 |

*Note.* *n* = 40.

**Point Biserial Correlation Analysis**

***Introduction***

A point biserial correlation analysis was conducted for dichotomic variables: gender, history of febrile convulsions (HFC), history of secondary generalized seizures (FBTCS), history of traumatic brain injury (TBI) and study variables. A point biserial correlation is a special case of the Pearson correlation. Cohen's standard was used to evaluate the strength of the relationships, where .1, .24, and .37 represent small, medium, and large effect sizes (Cohen, 1988). The result of the correlations was examined based on an alpha value of 0.05. Tables 7-10 present the results of the correlations.

**Table 9**

*Point Biserial Correlations for HFC and other study variables (LTLE)*

| Combination | *r*_pb_ | 95% CI | *p* |
| --- | --- | --- | --- |
| HFC-List-learning capacity | -0.35 | [-0.59, -0.06] | .243 |
| HFC-List-learning T1 | -0.41 | [-0.63, -0.12] | .096 |
| HFC-List-learning T5 | -0.27 | [-0.53, 0.02] | .791 |
| HFC-List-learning delayed | -0.31 | [-0.55, -0.01] | .506 |
| HFC-Story Memory | -0.11 | [-0.39, 0.20] | 1.000 |
| HFC-WM | -0.13 | [-0.41, 0.17] | 1.000 |
| HFC-Picture naming | -0.26 | [-0.52, 0.04] | .844 |
| HFC-Phonemic fluency | -0.06 | [-0.35, 0.24] | 1.000 |
| HFC-Semantic fluency | -0.22 | [-0.49, 0.08] | 1.000 |
| HFC-Education | -0.08 | [-0.37, 0.23] | 1.000 |
| HFC-Age | 0.17 | [-0.14, 0.44] | 1.000 |
| HFC-Age at onset | -0.03 | [-0.32, 0.27] | 1.000 |
| HFC-Epilepsy duration | 0.36 | [0.07, 0.59] | .223 |
| HFC-Frequency of seizures | 0.04 | [-0.26, 0.33] | 1.000 |
| HFC-AED number | 0.16 | [-0.15, 0.43] | 1.000 |

*Note. n = 44; HFC = history of febrile convulsions; LTLE = patients with left temporal lobe epilepsy; WM = working memory*

**Table 10**

*Point Biserial Correlations for HFC and other study variables (RTLE)*

| Combination | *r*_pb_ | 95% CI | *p* |
| --- | --- | --- | --- |
| HFC-List-learning capacity | 0.29 | [-0.11, 0.61] | 1.000 |
| HFC-List-learning T1 | 0.26 | [-0.14, 0.59] | 1.000 |
| HFC-List-learning T5 | 0.32 | [-0.07, 0.63] | 1.000 |
| HFC-List-learning delayed | 0.27 | [-0.13, 0.59] | 1.000 |
| HFC-Story Memory | -0.01 | [-0.39, 0.38] | 1.000 |
| HFC-WM | 0.12 | [-0.28, 0.49] | 1.000 |
| HFC-Picture naming | 0.24 | [-0.17, 0.57] | 1.000 |
| HFC-Phonemic fluency | 0.21 | [-0.19, 0.55] | 1.000 |
| HFC-Semantic fluency | 0.31 | [-0.08, 0.63] | 1.000 |
| HFC-Education | 0.05 | [-0.34, 0.43] | 1.000 |
| HFC-Age | -0.23 | [-0.56, 0.18] | 1.000 |
| HFC-Age at onset | -0.35 | [-0.65, 0.05] | 1.000 |
| HFC-Epilepsy duration | 0.21 | [-0.19, 0.55] | 1.000 |
| HFC-Frequency of seizures | -0.05 | [-0.43, 0.35] | 1.000 |
| HFC-AED number | 0.11 | [-0.29, 0.48] | 1.000 |

*Note. n = 26; HFC = history of febrile convulsions; RTLE = patients with right temporal lobe epilepsy; WM = working memory*

**Table 11**

*Point Biserial Correlations for* FBTCS *and other study variables (LTLE)*

| Combination | *r*_pb_ | 95% CI | *p* |
| --- | --- | --- | --- |
| FBTCS-List-learning capacity | -0.16 | [-0.44, 0.14] | 1.000 |
| FBTCS-List-learning T1 | -0.04 | [-0.33, 0.26] | 1.000 |
| FBTCS-List-learning T5 | -0.13 | [-0.41, 0.17] | 1.000 |
| FBTCS-List-learning delayed | -0.12 | [-0.40, 0.19] | 1.000 |
| FBTCS-Story Memory | -0.05 | [-0.34, 0.25] | 1.000 |
| FBTCS-WM | -0.06 | [-0.35, 0.24] | 1.000 |
| FBTCS-Picture naming | 0.18 | [-0.13, 0.45] | 1.000 |
| FBTCS-Phonemic fluency | 0.09 | [-0.22, 0.37] | 1.000 |
| FBTCS-Semantic fluency | -0.04 | [-0.33, 0.26] | 1.000 |
| FBTCS-Education | -0.03 | [-0.33, 0.27] | 1.000 |
| FBTCS-Age | 0.19 | [-0.11, 0.46] | 1.000 |
| FBTCS-Age at onset | 0.29 | [-0.01, 0.54] | .902 |
| FBTCS-Epilepsy duration | -0.06 | [-0.35, 0.24] | 1.000 |
| FBTCS-Frequency of seizures | 0.23 | [-0.07, 0.49] | 1.000 |
| FBTCS-AED number | -0.10 | [-0.39, 0.20] | 1.000 |

*Note. n = 44; FBTCS = history of secondary generalized seizures; LTLE = patients with left temporal lobe epilepsy; WM = working memory*

**Table 12**

*Point Biserial Correlations for FBTCS and other study variables (RTLE)*

| Combination | *r*_pb_ | 95% CI | *p* |
| --- | --- | --- | --- |
| FBTCS-List-learning capacity | 0.17 | [-0.23, 0.52] | 1.000 |
| FBTCS-List-learning T1 | 0.20 | [-0.20, 0.55] | 1.000 |
| FBTCS-List-learning T5 | 0.25 | [-0.15, 0.58] | 1.000 |
| FBTCS-List-learning delayed | 0.19 | [-0.21, 0.54] | 1.000 |
| FBTCS-Story Memory | 0.14 | [-0.26, 0.50] | 1.000 |
| FBTCS-WM | 0.03 | [-0.36, 0.41] | 1.000 |
| FBTCS-Picture naming | -0.02 | [-0.41, 0.37] | 1.000 |
| FBTCS-Phonemic fluency | 0.36 | [-0.03, 0.66] | 1.000 |
| FBTCS-Semantic fluency | 0.22 | [-0.19, 0.56] | 1.000 |
| FBTCS-Education | 0.14 | [-0.27, 0.50] | 1.000 |
| FBTCS-Age | 0.08 | [-0.32, 0.45] | 1.000 |
| FBTCS-Age at onset | 0.05 | [-0.35, 0.43] | 1.000 |
| FBTCS-Epilepsy duration | 0.04 | [-0.35, 0.42] | 1.000 |
| FBTCS-Frequency of seizures | 0.11 | [-0.29, 0.48] | 1.000 |
| FBTCS-AED number | -0.30 | [-0.62, 0.10] | 1.000 |

*Note. n = 26; FBTCS = history of secondary generalized seizures; RTLE = patients with right temporal lobe epilepsy; WM = working memory*

**Table 13**

*Point Biserial Correlations for TBI and other study variables (LTLE)*

| Combination | *r*_pb_ | 95% CI | *p* |
| --- | --- | --- | --- |
| TBI-List-learning capacity | 0.24 | [-0.06, 0.50] | .110 |
| TBI-List-learning T1 | 0.17 | [-0.14, 0.44] | .279 |
| TBI-List-learning T5 | 0.26 | [-0.04, 0.51] | .093 |
| TBI-List-learning Delayed | 0.12 | [-0.18, 0.40] | .435 |
| TBI-Story Memory | -0.00 | [-0.30, 0.29] | .979 |

*Note.* *n* = 44; TBI = history of traumatic brain injury

**Table 14**

*Point Biserial Correlations for TBI and other study variables (RTLE)*

| Combination | *r*_pb_ | 95% CI | *p* |
| --- | --- | --- | --- |
| TBI-List-learning capacity | 0.08 | [-0.32, 0.45] | .711 |
| TBI-List-learning T1 | 0.03 | [-0.36, 0.42] | .869 |
| TBI-List-learning T5 | 0.16 | [-0.24, 0.52] | .426 |
| TBI-List-learning Delayed | 0.16 | [-0.24, 0.52] | .427 |
| TBI-Story Memory | 0.10 | [-0.30, 0.47] | .629 |

*Note.* *n* = 26; TBI = history of traumatic brain injury

**Table 15**

*Point Biserial Correlations for gender and other study variables (LTLE)*

| Combination | *r*_pb_ | 95% CI | *p* |
| --- | --- | --- | --- |
| Gender-List-learning capacity | 0.14 | [-0.17, 0.42] | .377 |
| Gender-List-learning T1 | 0.21 | [-0.10, 0.47] | .180 |
| Gender-List-learning T5 | 0.07 | [-0.24, 0.36] | .673 |
| Gender-List-learning Delayed | 0.06 | [-0.24, 0.35] | .713 |
| Gender-Story Memory | 0.13 | [-0.17, 0.41] | .403 |

*Note.* *n* = 44;

**Table 16**

*Point Biserial Correlations for gender and other study variables (RTLE)*

| Combination | *r*_pb_ | 95% CI | *p* |
| --- | --- | --- | --- |
| Gender-List-learning capacity | 0.03 | [-0.37, 0.41] | .901 |
| Gender-List-learning T1 | -0.05 | [-0.43, 0.34] | .796 |
| Gender-List-learning T5 | -0.02 | [-0.41, 0.37] | .916 |
| Gender-List-learning Delayed | 0.05 | [-0.34, 0.43] | .802 |
| Gender-Story Memory | 0.00 | [-0.39, 0.39] | 1.000 |

*Note.* *n* = 26;

**Table 17**

*Point Biserial Correlations for gender and other study variables (Healthy Controls)*

| Combination | *r*_pb_ | 95% CI | *p* |
| --- | --- | --- | --- |
| Gender-List-learning capacity | 0.08 | [-0.24, 0.38] | .633 |
| Gender-List-learning T1 | -0.10 | [-0.40, 0.22] | .545 |
| Gender-List-learning T5 | 0.00 | [-0.31, 0.31] | 1.000 |
| Gender-List-learning Delayed | -0.02 | [-0.33, 0.30] | .917 |
| Gender-Story Memory | 0.10 | [-0.22, 0.40] | .532 |

*Note.* *n* = 40;

**Kendall Correlation Analysis**

***Introduction***

A Kendall correlation analysis was conducted among IQ levels and study variables. Cohen's standard was used to evaluate the strength of the relationships, where coefficients between .10 and .29 represent a small effect size, coefficients between .30 and .49 represent a moderate effect size, and coefficients above .50 indicate a large effect size (Cohen, 1988). The result of the correlations was examined based on an alpha value of 0.05. Tables 11-14 present the results of the correlations.

**Table 18**

*Kendall Correlation Results Among IQ level and study variables (LTLE)*

| Combination | *r*_k_ | 95% CI | *p* |
| --- | --- | --- | --- |
| IQ level-List-learning capacity | 0.45 | [0.18, 0.66] | .002 |
| IQ level-List-learning T1 | 0.33 | [0.03, 0.57] | .031 |
| IQ level-List-learning T5 | 0.52 | [0.26, 0.70] | < .001 |
| IQ level-List-learning delayed | 0.34 | [0.05, 0.58] | .023 |
| IQ level-Story Memory | 0.23 | [-0.07, 0.49] | .132 |
| IQ level-WM | 0.45 | [0.17, 0.66] | .002 |
| IQ level-Picture naming | 0.27 | [-0.03, 0.52] | .077 |
| IQ level-Phonemic fluency | 0.28 | [-0.02, 0.53] | .063 |
| IQ level-Semantic fluency | 0.29 | [-0.01, 0.54] | .056 |

*Note. n = 44. LTLE = patients with left temporal lobe epilepsy; WM = working memory*

**Table 19**

*Kendall Correlation Results Among IQ level and study variables (RTLE)*

| Combination | *r*_k_ | 95% CI | *p* |
| --- | --- | --- | --- |
| IQ level-List-learning capacity | 0.17 | [-0.23, 0.52] | .406 |
| IQ level-List-learning T1 | 0.01 | [-0.38, 0.40] | .949 |
| IQ level-List-learning T5 | 0.04 | [-0.35, 0.42] | .849 |
| IQ level-List-learning delayed | 0.19 | [-0.21, 0.54] | .356 |
| IQ level-Story Memory | 0.27 | [-0.13, 0.60] | .179 |
| IQ level-WM | 0.01 | [-0.38, 0.39] | .968 |
| IQ level-Picture naming | 0.19 | [-0.21, 0.54] | .342 |
| IQ level-Phonemic fluency | 0.48 | [0.12, 0.73] | .012 |
| IQ level-Semantic fluency | 0.33 | [-0.07, 0.64] | .099 |

*Note. n = 26. RTLE = patients with right temporal lobe epilepsy; WM = working memory*

**Table 20**

*Kendall Correlation Results Among IQ level and other socio-demographic and disease characteristics variables (LTLE)*

| Combination | *r*_k_ | 95% CI | *p* |
| --- | --- | --- | --- |
| IQ level-Education | 0.64 | [0.43, 0.79] | < .001 |
| IQ level-Age | 0.08 | [-0.22, 0.37] | .607 |
| IQ level-Age at onset | 0.14 | [-0.17, 0.42] | .379 |
| IQ level-Epilepsy duration | -0.13 | [-0.41, 0.17] | .404 |
| IQ level-Frequency of seizures | -0.11 | [-0.40, 0.19] | .467 |
| IQ level-AED number | -0.24 | [-0.50, 0.06] | .110 |

*Note. n = 44. LTLE = patients with left temporal lobe epilepsy*

**Table 21**

*Kendall Correlation Results Among IQ level and other socio-demographic and disease characteristics variables (LTLE)*

| Combination | *r*_k_ | 95% CI | *p* |
| --- | --- | --- | --- |
| IQ level-Education | 0.71 | [0.44, 0.86] | < .001 |
| IQ level-Age | 0.15 | [-0.25, 0.51] | .455 |
| IQ level-Age at onset | 0.15 | [-0.26, 0.50] | .478 |
| IQ level-Epilepsy duration | -0.14 | [-0.50, 0.26] | .483 |
| IQ level-Frequency of seizures | -0.05 | [-0.43, 0.34] | .797 |
| IQ level-AED number | 0.09 | [-0.31, 0.46] | .668 |

*Note. n = 26. RTLE = patients with right temporal lobe epilepsy*

**Table 22**

*Kendall Correlation Results Among IQ level and other study variables (Healthy Controls)*

| Combination | *r*_k_ | 95% CI | *p* |
| --- | --- | --- | --- |
| IQ level-List-learning capacity | 0.70 | [0.50, 0.83] | < .001 |
| IQ level-List-learning T1 | 0.61 | [0.37, 0.77] | < .001 |
| IQ level-List-learning T5 | 0.68 | [0.47, 0.82] | < .001 |
| IQ level-List-learning Delayed | 0.72 | [0.53, 0.84] | < .001 |
| IQ level-Story Memory | 0.66 | [0.45, 0.81] | < .001 |
| IQ level-Education | 0.77 | [0.60, 0.87] | < .001 |
| IQ level-Age | -0.34 | [-0.59, -0.03] | < .001 |

*Note.* *n* = 40.

**References**

Cohen, J. (1988). *Statistical power analysis for the behavior sciences* (2nd ed.). West Publishing Company.
