# Supplementary material for: Working Memory and Language Contribution to Verbal Learning and Memory in Drug-Resistant Unilateral Focal Temporal Lobe Epilepsy
Source: Front Neurol. 2021 Dec 8;12:780086. doi: 10.3389/fneur.2021.780086 (PMC8692669; doi:10.3389/fneur.2021.780086)
Supplement: Supplementary file 2 [file Table_2.DOCX]

***Supplementary Material 2***

**Summary of Hierarchical Regression Analysis for variables predicting verbal learning and memory in each participants’ group**

**Table 1**

*Summary of Hierarchical Regression Analysis for variables predicting verbal learning and memory (LTLE)*

| Variable | *B* | *SE* | 95% CI | β | *t* | *p* |
| --- | --- | --- | --- | --- | --- | --- |
| **List-learning capacity** |  |  |  |  |  |  |
| Step 1 |  |  |  |  |  |  |
| Education | 1.42 | 0.52 | [0.38, 2.46] | 0.36 | 2.75 | .009 |
| Age | -0.36 | 0.12 | [-0.61, -0.12] | -0.40 | -3.00 | .005 |
| Epilepsy duration | -0.10 | 0.14 | [-0.39, 0.19] | -0.10 | -0.72 | .475 |
| AED number | -2.90 | 1.70 | [-6.33, 0.54] | -0.24 | -1.71 | .096 |
| Step 2 |  |  |  |  |  |  |
| Education | 1.16 | 0.48 | [0.19, 2.14] | 0.30 | 2.41 | .021 |
| Age | -0.40 | 0.11 | [-0.62, -0.17] | -0.44 | -3.57 | .001 |
| Epilepsy duration | -0.10 | 0.13 | [-0.37, 0.17] | -0.10 | -0.76 | .452 |
| AED number | -1.40 | 1.65 | [-4.73, 1.94] | -0.11 | -0.85 | .402 |
| Working Memory | 0.91 | 0.32 | [0.26, 1.56] | 0.36 | 2.85 | .007 |
| Step 3 |  |  |  |  |  |  |
| Education | 1.03 | 0.47 | [0.07, 1.98] | 0.26 | 2.18 | .035 |
| Age | -0.42 | 0.11 | [-0.64, -0.20] | -0.47 | -3.90 | < .001 |
| Epilepsy duration | -0.08 | 0.13 | [-0.34, 0.17] | -0.08 | -0.66 | .515 |
| AED number | -1.54 | 1.59 | [-4.77, 1.68] | -0.13 | -0.97 | .339 |
| Working Memory | 0.77 | 0.32 | [0.13, 1.41] | 0.30 | 2.42 | .020 |
| Picture Naming | 1.01 | 0.52 | [-0.04, 2.05] | 0.23 | 1.95 | .058 |
| **List-learning Trial 1** |  |  |  |  |  |  |
| Step 1 |  |  |  |  |  |  |
| Epilepsy duration | -0.05 | 0.03 | [-0.12, 0.01] | -0.27 | -1.77 | .085 |
| AED number | -0.68 | 0.36 | [-1.41, 0.06] | -0.28 | -1.87 | .069 |
| Step 2 |  |  |  |  |  |  |
| Epilepsy duration | -0.05 | 0.03 | [-0.11, 0.00] | -0.27 | -2.00 | .052 |
| AED number | -0.29 | 0.35 | [-0.99, 0.42] | -0.12 | -0.83 | .414 |
| Working Memory | 0.22 | 0.07 | [0.09, 0.35] | 0.44 | 3.30 | .002 |
| **List-learning Trial 5** |  |  |  |  |  |  |
| Step 1 |  |  |  |  |  |  |
| Education | 0.44 | 0.14 | [0.15, 0.74] | 0.42 | 3.08 | .004 |
| AED number | -0.84 | 0.45 | [-1.75, 0.07] | -0.25 | -1.86 | .070 |
| Step 2 |  |  |  |  |  |  |
| Education | 0.38 | 0.14 | [0.09, 0.67] | 0.36 | 2.65 | .012 |
| AED number | -0.82 | 0.44 | [-1.70, 0.07] | -0.25 | -1.86 | .070 |
| Picture Naming | 0.30 | 0.16 | [-0.03, 0.62] | 0.25 | 1.86 | .070 |
| Step 3 |  |  |  |  |  |  |
| Education | 0.36 | 0.15 | [0.06, 0.65] | 0.34 | 2.44 | .019 |
| AED number | -0.67 | 0.46 | [-1.61, 0.27] | -0.20 | -1.44 | .159 |
| Picture Naming | 0.26 | 0.16 | [-0.07, 0.59] | 0.21 | 1.57 | .125 |
| Working Memory | 0.10 | 0.10 | [-0.11, 0.31] | 0.14 | 0.99 | .330 |
| **List-learning Delayed-recall** |  |  |  |  |  |  |
| Step 1 |  |  |  |  |  |  |
| Age | -0.11 | 0.04 | [-0.19, -0.02] | -0.35 | -2.52 | .016 |
| AED number | -1.25 | 0.57 | [-2.40, -0.11] | -0.30 | -2.21 | .033 |
| Step 2 |  |  |  |  |  |  |
| Age | -0.09 | 0.04 | [-0.18, -0.01] | -0.31 | -2.36 | .023 |
| AED number | -1.04 | 0.55 | [-2.14, 0.07] | -0.25 | -1.90 | .065 |
| Semantic Fluency | 0.19 | 0.08 | [0.03, 0.35] | 0.31 | 2.33 | .025 |
| **Story memory** |  |  |  |  |  |  |
| Step 1 |  |  |  |  |  |  |
| AED number | -1.87 | 0.80 | [-3.50, -0.25] | -0.34 | -2.33 | .025 |
| Step 2 |  |  |  |  |  |  |
| AED number | -1.58 | 0.79 | [-3.18, 0.02] | -0.29 | -1.99 | .053 |
| Semantic Fluency | 0.23 | 0.12 | [-0.01, 0.46] | 0.28 | 1.93 | .060 |

**Table 2**

*Summary of Linear / Hierarchical Regression Analysis for variables predicting verbal learning and memory (RTLE)*

| Variable | *B* | *SE* | 95% CI | β | *t* | *p* |
| --- | --- | --- | --- | --- | --- | --- |
| **List-learning capacity** |  |  |  |  |  |  |
| Picture Naming | 4.00 | 1.00 | [1.93, 6.06] | 0.63 | 4.00 | < .001 |
| **List-learning Trial 5** |  |  |  |  |  |  |
| Picture Naming | 0.61 | 0.22 | [0.16, 1.06] | 0.50 | 2.81 | .010 |
| **List-learning Delayed-recall** |  |  |  |  |  |  |
| Step 1 |  |  |  |  |  |  |
| Age | -0.11 | 0.07 | [-0.24, 0.03] | -0.31 | -1.61 | .120 |
| Step 2 |  |  |  |  |  |  |
| Age | -0.10 | 0.06 | [-0.22, 0.02] | -0.30 | -1.77 | .090 |
| Picture Naming | 1.00 | 0.36 | [0.26, 1.73] | 0.48 | 2.79 | .010 |
| **Story memory** |  |  |  |  |  |  |
| Step 1 |  |  |  |  |  |  |
| Education | 0.63 | 0.43 | [-0.26, 1.53] | 0.28 | 1.47 | .156 |
| Age at onset | 0.12 | 0.08 | [-0.04, 0.28] | 0.31 | 1.60 | .123 |
| Step 2 |  |  |  |  |  |  |
| Education | 0.60 | 0.32 | [-0.06, 1.26] | 0.27 | 1.89 | .072 |
| Age at onset | 0.09 | 0.06 | [-0.03, 0.21] | 0.22 | 1.56 | .133 |
| Working Memory | 0.83 | 0.18 | [0.45, 1.21] | 0.62 | 4.52 | < .001 |
| Step 3 |  |  |  |  |  |  |
| Education | 0.57 | 0.38 | [-0.23, 1.36] | 0.25 | 1.47 | .155 |
| Age at onset | 0.09 | 0.06 | [-0.03, 0.22] | 0.23 | 1.51 | .145 |
| Working Memory | 0.82 | 0.21 | [0.39, 1.25] | 0.61 | 3.97 | < .001 |
| Semantic Fluency | 0.02 | 0.14 | [-0.27, 0.32] | 0.03 | 0.18 | .862 |

**Table 3**

*Summary of Hierarchical Regression Analysis for variables predicting verbal learning and memory (Healthy Controls)*

| Variable | *B* | *SE* | 95% CI | β | *t* | *p* |
| --- | --- | --- | --- | --- | --- | --- |
| **List-learning capacity** |  |  |  |  |  |  |
| Step 1 |  |  |  |  |  |  |
| Age | -0.32 | 0.08 | [-0.48, -0.15] | -0.54 | -3.93 | < .001 |
| Step 2 |  |  |  |  |  |  |
| Age | -0.17 | 0.08 | [-0.32, -0.02] | -0.29 | -2.28 | .029 |
| Working Memory | 1.03 | 0.24 | [0.54, 1.52] | 0.54 | 4.23 | < .001 |
| Step 3 |  |  |  |  |  |  |
| Age | -0.14 | 0.07 | [-0.28, 0.01] | -0.23 | -1.86 | .071 |
| Working Memory | 0.65 | 0.27 | [0.11, 1.19] | 0.34 | 2.44 | .020 |
| Picture Naming | 0.22 | 1.31 | [-2.45, 2.88] | 0.02 | 0.17 | .870 |
| Phonemic Fluency | 0.64 | 0.23 | [0.18, 1.10] | 0.38 | 2.81 | .008 |
| **List-learning Trial 1** |  |  |  |  |  |  |
| Step 1 |  |  |  |  |  |  |
| Age | -0.04 | 0.02 | [-0.07, -0.01] | -0.37 | -2.43 | .020 |
| Step 2 |  |  |  |  |  |  |
| Age | -0.01 | 0.02 | [-0.04, 0.02] | -0.12 | -0.81 | .424 |
| Working Memory | 0.18 | 0.05 | [0.08, 0.28] | 0.54 | 3.72 | < .001 |
| Step 3 |  |  |  |  |  |  |
| Age | -0.01 | 0.02 | [-0.04, 0.02] | -0.12 | -0.78 | .442 |
| Working Memory | 0.14 | 0.06 | [0.03, 0.25] | 0.42 | 2.54 | .016 |
| Picture Naming | -0.26 | 0.27 | [-0.82, 0.29] | -0.16 | -0.96 | .346 |
| Phonemic Fluency | 0.11 | 0.05 | [0.02, 0.21] | 0.38 | 2.41 | .021 |
| **List-learning Trial 5** |  |  |  |  |  |  |
| Step 1 |  |  |  |  |  |  |
| Age | -0.04 | 0.01 | [-0.06, -0.01] | -0.39 | -2.58 | .014 |
| Step 2 |  |  |  |  |  |  |
| Age | -0.02 | 0.01 | [-0.05, 0.01] | -0.25 | -1.66 | .105 |
| Picture Naming | -0.22 | 0.25 | [-0.72, 0.29] | -0.15 | -0.88 | .387 |
| Phonemic Fluency | 0.15 | 0.04 | [0.07, 0.24] | 0.56 | 3.59 | < .001 |
| Step 3 |  |  |  |  |  |  |
| Age | -0.02 | 0.01 | [-0.05, 0.01] | -0.19 | -1.28 | .210 |
| Picture Naming | -0.39 | 0.25 | [-0.89, 0.12] | -0.26 | -1.55 | .129 |
| Phonemic Fluency | 0.12 | 0.04 | [0.03, 0.21] | 0.44 | 2.78 | .009 |
| Working Memory | 0.11 | 0.05 | [0.01, 0.21] | 0.37 | 2.20 | .035 |
| **List-learning Delayed-recall** |  |  |  |  |  |  |
| Step 1 |  |  |  |  |  |  |
| Age | -0.07 | 0.02 | [-0.11, -0.03] | -0.46 | -3.22 | .003 |
| Step 2 |  |  |  |  |  |  |
| Age | -0.04 | 0.02 | [-0.08, 0.00] | -0.26 | -1.87 | .070 |
| Picture Naming | -0.02 | 0.36 | [-0.75, 0.72] | -0.01 | -0.05 | .961 |
| Phonemic Fluency | 0.24 | 0.06 | [0.12, 0.37] | 0.56 | 3.90 | < .001 |
| Step 3 |  |  |  |  |  |  |
| Age | -0.03 | 0.02 | [-0.07, 0.01] | -0.20 | -1.47 | .151 |
| Picture Naming | -0.29 | 0.36 | [-1.02, 0.43] | -0.12 | -0.82 | .419 |
| Phonemic Fluency | 0.19 | 0.06 | [0.06, 0.31] | 0.44 | 3.06 | .004 |
| Working Memory | 0.18 | 0.07 | [0.03, 0.33] | 0.37 | 2.48 | .018 |
| **Story Memory** |  |  |  |  |  |  |
| Step 1 |  |  |  |  |  |  |
| Age | -0.09 | 0.04 | [-0.17, -0.00] | -0.32 | -2.07 | .045 |
| Step 2 |  |  |  |  |  |  |
| Age | 0.00 | 0.04 | [-0.07, 0.07] | 0.01 | 0.09 | .927 |
| Working Memory | 0.64 | 0.11 | [0.41, 0.88] | 0.72 | 5.65 | < .001 |
| Step 3 |  |  |  |  |  |  |
| Age | 0.00 | 0.03 | [-0.07, 0.07] | 0.01 | 0.08 | .938 |
| Working Memory | 0.55 | 0.13 | [0.29, 0.81] | 0.62 | 4.30 | < .001 |
| Picture Naming | -0.75 | 0.62 | [-2.01, 0.52] | -0.17 | -1.20 | .240 |
| Phonemic Fluency | 0.29 | 0.11 | [0.08, 0.51] | 0.37 | 2.73 | .010 |
